# Proximal hip fractures in 71,920 elderly patients: incidence, epidemiology, mortality and costs from a retrospective observational study

**DOI:** 10.1186/s12889-023-16776-4

**Published:** 2023-10-10

**Authors:** Marco Viganò, Federico Pennestrì, Elisabetta Listorti, Giuseppe Banfi

**Affiliations:** 1 IRCCS Istituto Ortopedico Galeazzi, Via Cristina Belgioioso 173, Milan, 20157 Italy; 2 Centre for Healthcare and Social Care Management (CERGAS), SDA Bocconi, Milan, 20136 Italy; 3 Vita-Salute San Raffaele University, Via Olgettina 58, Milan, 20132 Italy

**Keywords:** Elderly, Healthcare policy, Lombardy, Mortality, Osteoporosis, Proximal femoral fracture, Prevention, Social determinants of health, Value-based care

## Abstract

**Background:**

The risk of proximal femoral fractures increases with aging, causing significant morbidity, disability, mortality and socioeconomic pressure. The aims of the present work are (1) to investigate the epidemiology and incidence of these fractures among the elderly in the Region of Lombardy; (2) to identify the factors influencing survival; (3) to identify the factors influencing hospitalization and post-operative costs.

**Methods:**

The Region of Lombardy provided anonymized datasets on hospitalized patients with a femoral neck fracture between 2011 and 2016, and anonymized datasets on extra-hospital treatments to track the patient history between 2008 and 2019. Statistical evaluations included descriptive statistics, survival analysis, Cox regression and multiple linear models.

**Results:**

71,920 older adults suffered a femoral fracture in Lombardy between 2011 and 2016. 76.3% of patients were females and the median age was 84. The raw incidence of fractures was stable from year 2011 to year 2016, while the age-adjusted incidence diminished. Pertrochanteric fractures were more spread than transcervical fractures. In patients treated with surgery, receiving treatment within 48 h reduced the hazard of death within the next 24 months. Combined surgical procedures led to increased hazard in comparison with arthroplasty alone, while no differences were observed between different arthroplasties and reduction or fixation. In patients treated conservatively, age and male gender were associated with higher hazard of death. All patients considered, the type of surgery was the main factor determining primary hospitalization costs. A higher number of surgeries performed by the index hospital in the previous year was associated with financial savings. The early intervention significantly correlated with minor costs.

**Conclusions:**

The number of proximal femoral fractures is increasing even if the age-adjusted incidence is decreasing. This is possibly due to prevention policies focused on the oldest cohort of the population. Two policies proved to be significantly beneficial in clinical and financial terms: the centralization of patients in high-volume hospitals and a time limit of 48 h from fracture to surgery.

**Trial registration:**

Non applicable.

## Background

Proximal femoral fractures are common among elderly patients and cause significant morbidity, disability, mortality and socioeconomic pressure on healthcare systems and caregivers worldwide [1–5]. These fractures are expected to reach up to 21.3 million globally by 2050 as a consequence of aging [6], bone mass decrease and higher chances of accidental falls [7]. The incidence of proximal femoral fractures among people aged 65 or older varies in different countries depending on age itself, sex, comorbidities and lifestyle, reaching a peak among patients 85–89 years old, affecting more women than men and being exacerbated by cognitive disfunction and institutionalisation [8–10]. The direct costs of hip fracture treatment have a major economic impact on the healthcare system worldwide [7, 11, 12], not to mention the indirect impact on caregivers and society more in general which is under investigated.

While in Italy the absolute number of these events increases, the incidence adjusted per age seems to decrease [8], possibly as a consequence of beneficial prevention policies adopted by those regions where aging is specifically addressed. Examples are preventive osteoporosis treatment, fall prevention policies inside healthcare facilities and promotion of a more active lifestyle to improve general health and frailty [13].

The objectives of the present work are: (1) to describe the epidemiology and incidence of proximal femur fractures (number of patients, number of fractures, type of fractures, population age, sex and comorbidities) among the elderly in Lombardy, the Region with the highest population in Italy, the highest active mobility (patients travelling from other Italian Regions to be cured in a local hospital) and a substantial amount of elderly patients [14]; (2) to identify the factors influencing survival (patients’ characteristics, type of treatment, hospital volumes, time to surgery); (3) to identify the factors influencing the costs borne by the Regional healthcare system, including hospitalization and post-discharge care. Overall, we aim to underline some key factors to improve the management of similar patients in similar healthcare systems, which is necessary to reduce the human, societal and financial burden of femoral fractures in aging countries.

## Methods

### Data source

Data were retrieved from the healthcare information system of the Region of Lombardy. IRCCS Istituto Ortopedico Galeazzi is an official partner of the Region of Lombardy for the analysis of healthcare data as per Regional Decree n.10,403 (August 30th, 2017).

The information system contains data on service provided to regional citizens such as:

- outpatient pharmaceutical prescriptions,
- specialistic visits and diagnostic examinations,
- access to emergency department,
- hospital admissions.

The drug prescriptions are coded according to the Anatomical Therapeutic Chemical (ATC) classification system; inpatient diagnoses, procedures and outpatient visits are coded according to the International Classification of Disease, Ninth Revision, Clinical Modification (ICD-9-CM). Thanks to a personal identification code it is possible to link each treatment into a complete care pathway. In order to preserve privacy, identification codes are automatically converted into anonymous codes, and the inverse process is prevented by deletion of the conversion table.

In Italy, analyses of an anonymous administrative database do not require Ethic Committee approval.

### Study cohort

This is a retrospective population-based cohort study. The Region of Lombardy provided anonymized data on hospitalized patients with a femoral neck fracture between January 1st, 2011 and December 31st, 2016, identified from the hospital discharge records reporting the ICD codes 820.0-820.9. The first fracture within the time range under investigation was considered the patient’s index hospitalization. The final cohort included patients older than 65 years old without polytraumas.

Then, we retrieved data on previous injuries and pharmacological therapies which may have influenced the fracture occurrence from January 1st, 2008 to June 30th, 2019.

**Fig. 1:**
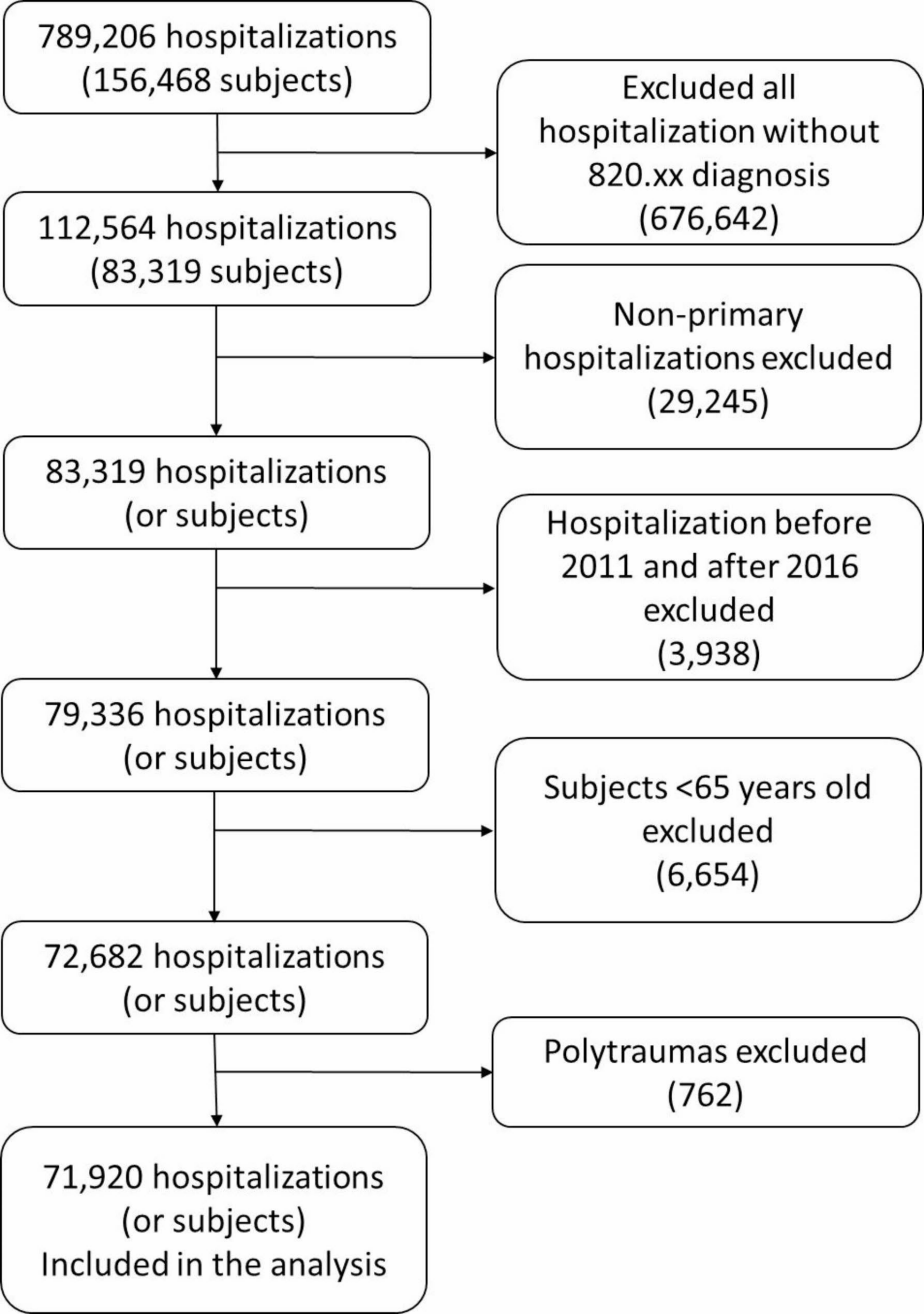
Patients’ selection flow chart

The patient selection process is represented by the flow chart in Fig. 1.

Each patient follow-up started with the index hospitalisation to end 24 months after.

### Costs

All the costs covered by the Region of Lombardy were collected and aggregated into specific time-periods (3, 6, 12, 18, 24 months from index hospitalization onwards).

### Statistical methods

The analyses were performed using R software v4.1.0 [15].

Categorical variables are reported as absolute frequencies and percentages, while continuous data are reported as medians and interquartile range (IQR), unless otherwise specified. Raw incidence rate per 100,000 person-years is reported. Age-adjusted incidence was calculated by direct standardization of the population by year per age category. Differences in proportions were assessed using Chi-squared tests or Fisher’s exact test.

Kaplan-Meier curves were calculated for the whole population and for the subgroups of interest; differences were tested by the log-rank test.

Cox regression models were selected by a stepwise process starting from a full model aimed to maximize likelihood.

Hazard Ratios were calculated exponentiating the coefficients obtained from Cox models.

Multiple linear regression models were used to assess the effect of specific covariates and hospitalization costs (continuous). Models’ selection was performed by a stepwise backward process based on AIC minimization.

## Results

### Population: number of patients, age, sex and comorbidities

A total of 71,920 older adults with a femoral neck fracture in Lombardy between 2011 and 2016 were identified. The large majority of patients were females (54,877, 76.3%) and the median age was 84 years (interquartile range: 79–89). Females were slightly older than males (85 vs. 83 years old in median, p < 0.001). These characteristics are summarized in Table 1.

**Table 1:** Patients’ characteristics

	Total	Males	Females
Primary Events	71,920*	17,040	54,877
Age	84 (79–89)	83 (77–88)	85 (79–89)

*Including 3 subjects who did not report their sex. Data reported as absolute frequency (and column percentage if appropriate) or median (and interquartile range)

### Number of fractures and incidence

**Fig. 2:**
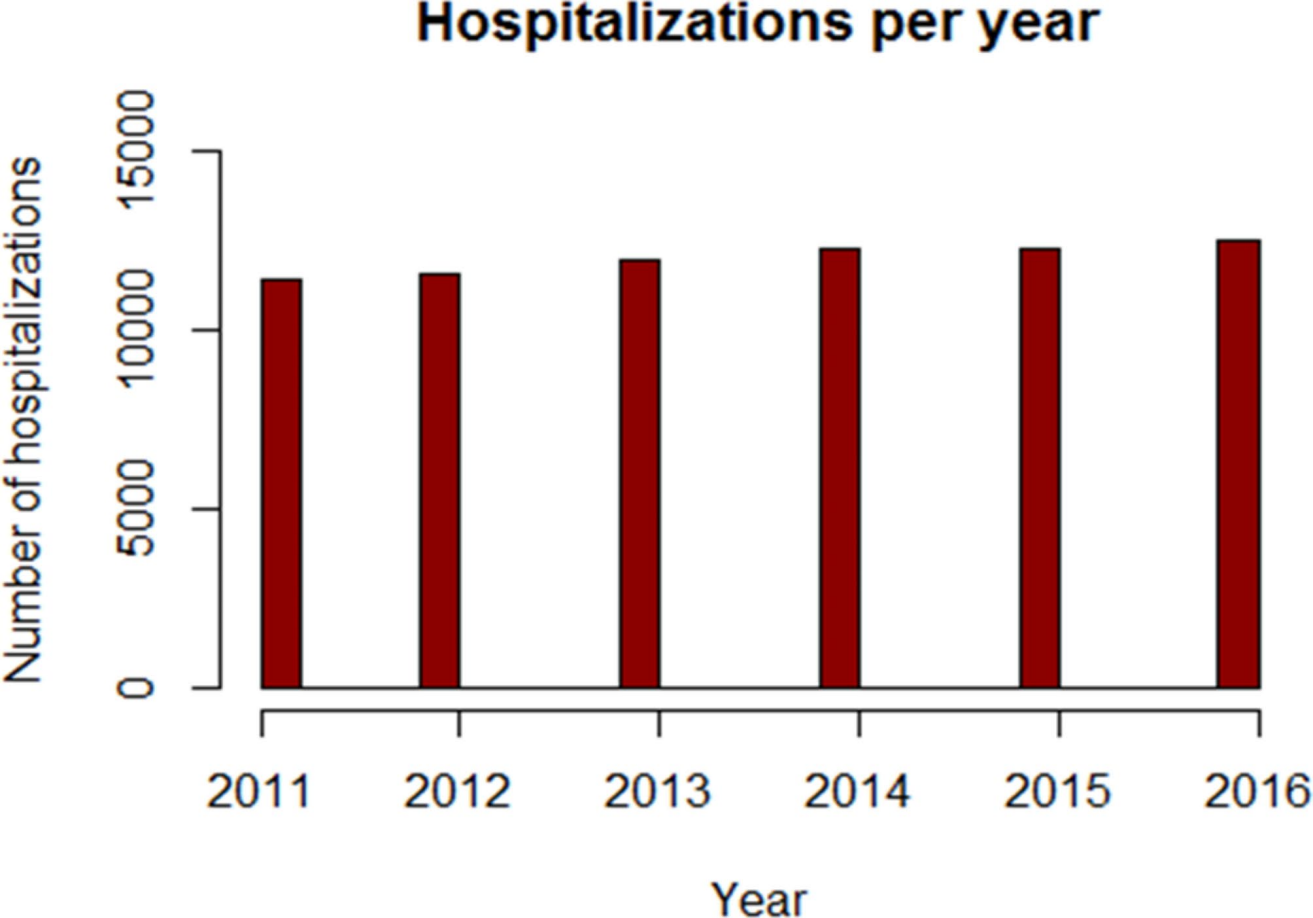
Absolute number of hospitalizations per femoral neck fractures, in Lombardy, in patients ≥ 65 years

The total number of hospitalizations for fracture of the femoral neck, in the Region of Lombardy, between 2011 and 2016, was 71,920 in patients aged 65 years or more. The mean raw incidence rate was 537,1 per 100,000 person-years, while the mean age-adjusted incidence was 574,6 per 100,000 person-years. The hospitalization number grew constantly from 11,420 to 12,508 (+ 9,5%), as illustrated by Fig. 2.

**Fig. 3:**
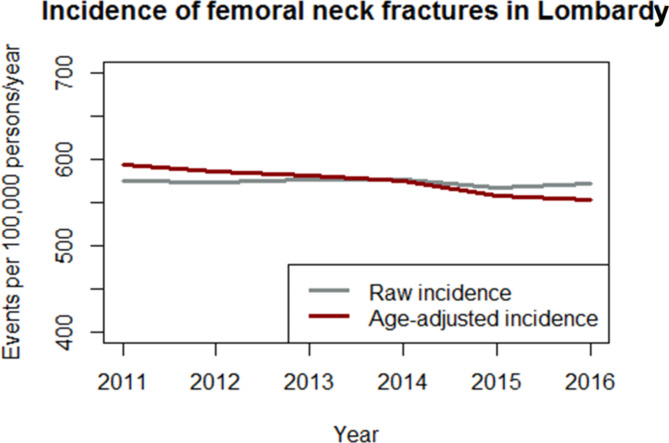
Incidence of femoral neck fractures in Lombardy, raw and adjusted per age

The raw incidence of fractures was substantially stable from year 2011 to year 2016, while the incidence adjusted per age diminished from 593,7 events per 100,000 person-years to 553,6 (-6,8%), as illustrated by Fig. 3.

#### Type of fractures

**Fig. 4:**
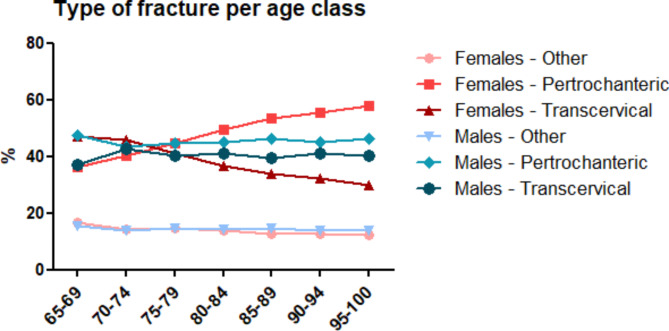
Type of fracture per age class

Pertrochanteric fractures were more frequent (49.2%) than transcervical fractures (37.5%) in the whole group and equally spread between sexes. Other/Unspecified type of fractures (13.3%) were observed. Fracture type varied significantly depending on age class in female patients, while no association was observed in males. Pertrochanteric fractures in younger females (65–69 y.o.) represented the 36.1% and increased up to 57.9% in the 95–100 age group. On the contrary, transcervical fractures represented the 47.2% among 65–69 y.o. females and decreased to 30.0% in the oldest age class. Open fractures were rare events (1.9%) as most of the observed fractures were closed (98.1%). These data are illustrated by Fig. 4.

### Factors influencing survival

**Fig. 5:**
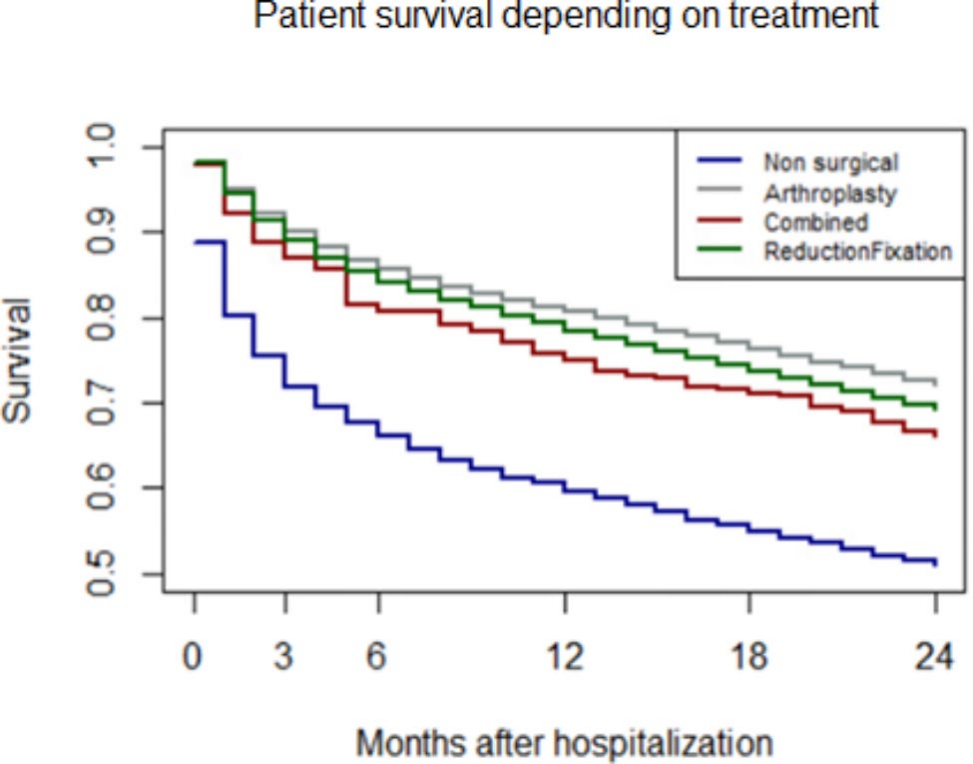
Patient survival depending on treatment, up to two years

The extreme survival variation between patients who underwent surgery (89.4%) and those treated conservatively (10.6%) (Fig. 5) did not allow to obtain a Cox regression model consistent with the modelling assumptions, in particular with proportional hazards. Then, two different models were produced: one representing the patients who underwent surgery and one representing the patients treated conservatively.

In patients treated with surgery, receiving treatment within 48 h reduced the hazard of death in the first 3 months after hospitalization by 19.5%, while the difference reached 26.5% between 3 and 9 months and diminished to 12.6% between 9 and 24 months. The hazard reduction was significantly different between the periods 3–9 and 9–24 months (Confidence Interval 95% 0.69–0.77 vs. 0.83-0.92). Hazard varied significantly also depending on gender, with males’ hazard 2.44 times higher than females in the first 3 months after hospitalization, progressively decreasing to 2.10 at 3–9 months and reaching 1.86 times the hazard of females in the next 15 months. Combined surgical procedures (arthroplasty plus reduction or fixation) led to increased hazard (+ 33%) in comparison with arthroplasty alone, while no differences were observed between different arthroplasties and reduction or fixation. Increased hazard per year of age was 7.6%. In terms of hospital characteristics, a constant hazard increase was observed per 1% surgeries performed within 48 h in the previous year, equal to + 12.8%; for each hospitalization with a diagnosis of proximal femoral fracture, the risk of death decreased by 0.002% in the first period, and by 0.003% between 3 and 24 months. These findings are reported in Table 2.

**Table 2:** Results of the Cox regression model for survival in patients treated surgically

	coef	exp(coef)	se(coef)	Lower bound CI95%	Upper bound CI95%	P value
age	0.073	1.076	0.001	1.073	1.078	< 0.001
Fracture Type:Pertrochanter	0.024	1.024	0.032	0.961	1.091	0.464
Fracture Type:Transcervical	-0.082	0.921	0.024	0.878	0.966	0.001
Surgery:Combined	0.287	1.333	0.112	1.069	1.661	0.011
Surgery:Reduction Fixation	0.042	1.042	0.027	0.989	1.099	0.125
Percentage of surgeries within 48 h	0.120	1.128	0.040	1.042	1.221	0.003
genderFemale:strata = 1	-0.892	0.410	0.025	0.390	0.430	< 0.001
genderFemale:strata = 2	-0.743	0.476	0.031	0.448	0.505	< 0.001
genderFemale:strata = 3	-0.624	0.536	0.026	0.509	0.564	< 0.001
strata = 1:number of hospitalizations for PFF	-0.0002	0.9998	0.0001	0.9997	1.0000	0.012
Strata = 2: number of hospitalizations for PFF	-0.0003	0.9997	0.0001	0.9996	0.9999	0.000
strata = 3: number of hospitalizations for PFF	-0.0003	0.9997	0.0001	0.9995	0.9998	0.000
strata = 1: delay < 48 h	-0.217	0.805	0.025	0.766	0.846	< 0.001
strata = 2: delay < 48 h	-0.308	0.735	0.029	0.694	0.779	< 0.001
strata = 3: delay < 48 h	-0.135	0.874	0.024	0.833	0.916	< 0.001

Strata: temporal period (1 = 0 to 3 months; 2 = 3 to 9 months, 3 = 9 to 24 months). PFF = proximal femoral fractures. Reference categories: Fracture Type = Other; Surgery = Arthroplasties; gender = Male; delay = > 48 h

In patients treated conservatively, age (+ 5.5% per year) and male gender (+ 96.8%) were associated with higher hazard of death. The number of hospitalizations per hospital reduced the risk by 0.06% per unit, while the total number of surgeries per hospital increased it by + 0.04% per unit. Patients with pertrochanteric fractures had lower hazard of death in the second time period (3–24 months) compared to patients with transcervical fractures (-20.0%). The percentage of surgeries within 48 h was associated with a significantly lower hazard of death (per 1%) in the first period (-62.6%, CI95%: -69.2%, -54.6%) in comparison to the second (-31.3%, CI95%: -44.9%, -14.4%). The results are reported in Table 3.

**Table 3:** Results of the Cox regression model for survival in patients treated conservatively

	coef	exp(coef)	se(coef)	lower CI95%	upper CI95%	P value
age	0.054	1.055	0.002	1.050	1.060	< 0.001
genderMale	0.677	1.968	0.034	1.840	2.104	< 0.001
Number of surgeries for PFF	0.000	1.000	0.000	1.000	1.001	0.002
Number of hospitalizations for PFF	-0.001	0.999	0.000	0.999	1.000	0.000
FractureType Other:strata = 1	0.008	1.008	0.060	0.897	1.133	0.888
FractureType Pertrochanter:strata = 1	-0.040	0.961	0.050	0.871	1.059	0.418
Fracture type Other:strata = 2	0.083	1.086	0.067	0.953	1.239	0.216
Fracture type Pertrochanter:strata = 2	-0.223	0.800	0.059	0.712	0.898	0.000
Percentage surgeries within48h:strata = 1	-0.983	0.374	0.099	0.308	0.454	< 0.001
Percentage surgeries within48h:strata = 2	-0.376	0.687	0.112	0.551	0.856	0.001

Strata: temporal period (1 = 0 to 3 months; 2 = 3 to 24 months). PFF = proximal femoral fractures. Reference categories: Fracture Type = Transcervical; gender = Female

### Factors influencing hospitalization costs

All patients considered, type of surgery was the main factor determining primary hospitalization costs. Compared to non-surgical treatments, arthroplasty increased costs by a mean of €5.138 (Standard Error, SE, €36), while reduction/fixation increased costs by €1.938 (SE €37). The choice of treatment is primarily defined by the type of fracture, with pertrochanteric localization usually treated by reduction or fixation and transcervical fractures treated by hemiarthroplasty or total hip arthroplasty (THA). Then, adjustments were needed on these estimations based on type of fracture. Male patients were more costly than females (+€90, SE €22) per hospitalization, while age had little and non-significant effects on costs.

A higher number of surgeries performed by the hospital was associated with financial savings. In particular, for each 1,000 surgeries in the year before the index hospitalization, costs were reduced by €764 (SE €67), while the total number of admissions per hospital in the year before the event increased costs by €189 (SE €85) per 1.000 cases. Hospitals with higher percentage of surgeries within 48 h demonstrated a non-significant cost reduction of €72 (SE €50) per %unit, suggesting a possible role for this parameter in improving efficiency. These results are reported in Table 4.

**Table 4:** Factors influencing hospitalization costs in the whole cohort

	Estimate	Std.Error	t value	Pr(>|t|)
(Intercept)	4334.72	123.77	35.02	< 0.001
age	-2.28	1.35	-1.69	0.091
Gender:Male	90.297	22.92	3.94	< 0.001
Surgtype:Arthroplasty	5138.17	36.11	142.31	< 0.001
Surgtype:Combined	5785.26	38.73	33.73	< 0.001
Surgtype:ReductionFixation	1938.89	36.65	52.91	< 0.001
Fracture type: Pertrochanteric	155.95	38.73	4.03	< 0.001
Fracture type: Transcervical	-249.39	31.80	-7.84	< 0.001
Number of surgeries for PFF	-0.76	0.06	-11.36	< 0.001
Percentage surgeries within48h	-71.72	49.65	-1.44	0.149
Number of hospitalizations for PFF	0.19	0.09	2.20	0.028

PFF = proximal femoral fractures. Reference categories: Fracture Type = Other; gender = Female

Considering only patients treated surgically, the early intervention was significantly correlated with minor costs, with a mean of €325 (SE €20). Male gender (+€106, SE €22) and arthroplasty (+€3.141, SE €33.7) were confirmed to increase costs. In this cohort, older age was significantly associated with increased costs (+€9 per year), while the number of proximal femoral fracture hospitalizations and percentage of interventions within 48 h per hospital reduced costs significantly (-€572 per 1.000 hospitalizations and -€330 per %unit, respectively). These findings are reported in Table 5.

**Table 5:** Factors influencing hospitalization costs in patients treated surgically

	Estimate	Std.Error	t value	P value
(Intercept)	9,12E + 06	1,17E + 05	78.17	< 0,001
age	3,08E + 03	1,31E + 03	2.35	0.019
Gender: Male	1,06E + 05	2,25E + 04	4.70	< 0,001
Surgery: Combined	6,73E + 05	1,56E + 05	4.32	< 0,001
Surgery: ReductionFixation	-3,14E + 06	3,37E + 04	-93.20	< 0,001
Fracture type: Pertrochanteric	2,88E + 05	4,09E + 04	7,04E + 03	< 0,001
Fracture type: Transcervical	-2,27E + 04	3,17E + 04	-0.72	0.474
Number of surgeries for PFF	-1,04E + 02	6,58E + 01	-1.58	0.115
Percentage surgeries within48h	-3,30E + 05	5,29E + 04	-6.24	< 0,001
Number of hospitalizations for PFF	-5,74E + 02	8,39E + 01	-6.82	< 0,001
delay < 48 h	-3,25E + 05	2,04E + 04	-15.92	< 0,001

PFF = proximal femoral fractures. Reference categories: Fracture Type = Other; Surgery = Arthroplasty; gender = Female, delay = > 48 h

**Fig. 6:**
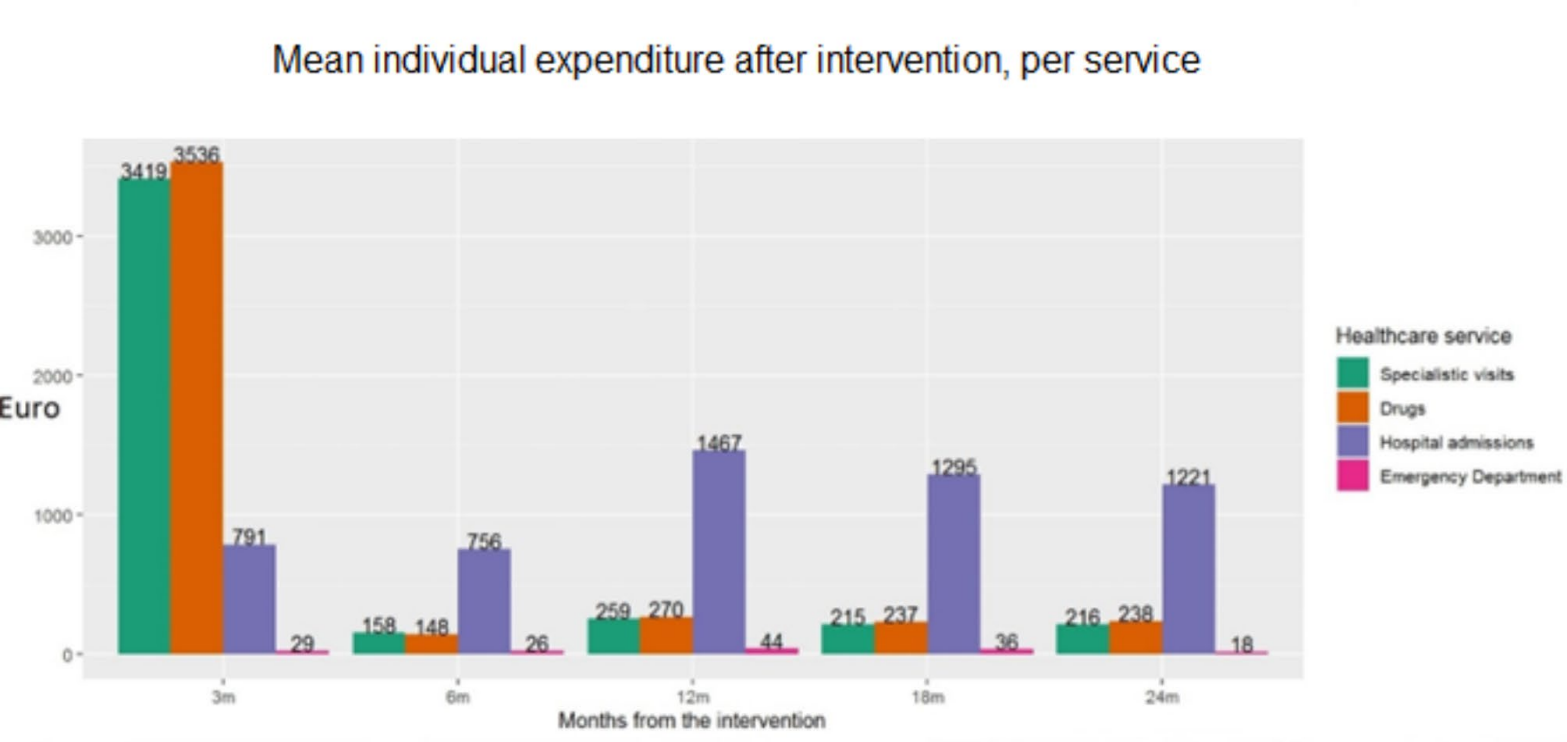
Mean individual expenditure after the intervention, per service (only patients alive at the beginning of the time-period were considered)

No significant association between earlier intervention and costs after first discharge was observed. Pharmacological treatment and outpatient care (in particular rehabilitation) represent most of the costs covered by the Region within 3 months after discharge, while the largest share in the following time sessions was represented by new hospitalizations, both related and non-related to the index admission (Fig. 6).

## Discussion

The raw incidence of femoral fractures among patients aged 65 or more remained almost stable in the period under investigation, while the age-adjusted percentage was reduced by more than 100 cases per 100.000 inhabitants per year. These findings suggest the beneficial effects of a major focus on the elderly population as exemplified in the background session. These observations are consistent with those reported by authors from other Regions of the world [16, 17].

Most of the international literature, guidelines [18] and Italian data on the epidemiology and management of proximal femur fractures were confirmed by data from the Region of Lombardy: the higher frequency of females and patients older than 80 years, the higher prevalence of pertrochanteric fractures over transcervical ones, internal fixation more common to treat pertrochanteric fractures and arthroplasty (both total and partial) more common to treat transcervical fractures [8]. For example, the adoption of THA to fix transcervical fractures is consistent with reports from other countries like Australia, Canada, Finland, South Korea and United States [19–24]. The same is recommended by the American Academy of Orthopaedic Surgeons practical guidelines, especially for younger patients, since there is no evidence of higher effectiveness of THA over hemi hip arthroplasty in older individuals [18, 25].

Besides type of fracture and age, proximal femoral fractures management may be influenced by the surgeon’s expertise, the number of procedures performed at the index hospital and the presence of patient insurance, as patients covered by a private insurance undergo THA more frequently [26, 27]. This study shows an extremely high impact of treatment choice on patient survival, in particular the higher mortality of patients treated conservatively. Indeed, the decision in favor of surgical over a conservative treatment is mostly based on the general condition of the patient [28]. Thus, it is not possible to attribute increased mortality to the specific therapeutic choice, since it is not independent to the risk of death, that is the main reason beyond this decision. Nonetheless, another factor that could lead to a non-surgical management is the experience of the clinical team with “borderline” patients who may be left untreated more often in unspecialized centers.

Then, the centralization of patients in highly specialized hospital is a valuable opportunity for policymakers. In our series, patients treated in higher volume centres reported a lower hazard of death within the following 24 months. In addition, higher hospital volumes resulted cheaper than centers reporting a lower admission number, once adjusted for the type of treatment (the main driver of cost changes). Therefore the centralization of interventions may both improve treatment outcomes and reduce the costs sustained by the healthcare system. The difference between local and specialized centers may appear obvious in countries characterised by relevant geographic separation between rural and urban areas, such the US or Australia [29, 30], but may be surprising for European countries where the distance between differently populated region is limited and a number of clinics is distributed on the territory: the Region of Lombardy is the perfect example [14]. Nevertheless, the importance of centralization emerged clearly from these analyses.

Another important policy for the improvement of proximal femoral fractures management was the inclusion of 48 h time to surgery among the high standards of care. In the population of interest, a surgical intervention within 48 h was correlated to significant benefit in terms of chances of survival at 24 months, especially in the first months after the event, also reducing costs significantly.

Of course, the 48 h limit represents a compromise between system capacity and patients’ benefit. Other countries, like Sweden, fixed this limit to 24 h [31]. Further reductions below 24 h were not associated with improvements in patients’ survival, as demonstrated by a multicenter randomized clinical trial testing 6 vs. 24 h time-limits [32].

Another factor strongly associated with 2-year mortality is gender, with male patients facing a significantly higher risk of death when compared to females. The analysis reported in the present study demonstrate that this effect remained relevant and significant even after adjustment for age, thus no obvious explanation can be provided. Interestingly, other authors confirmed the higher mortality of male patients after controlling for age and health status [33]. The results of the Baltimore Hip Study identified infections (pneumonia, influenza, and septicemia) as the main causes of excessive deaths in men after hip fractures [34], but the reasons behind the higher incidence of infections-related deaths in males are still unknown. This difference between genders may also explain the reduced risk of death in patients affected by pertrochateric fractures, as these patients are more frequently females, especially in old age [8].

Not only were hospital centralization, timely surgical intervention and female gender associated with reduced risk of death, but also with overall cost reduction per admission. Besides length of stay it was not possible to evaluate specific sources of cost avariation as they were not available in the database.

This study has some limitations. The reliance on administrative data collected retrospectively do not allow for a complete evaluation of clinical outcomes, such as relieve from pain and recovery of functional activity. In addition, we could not retrieve data on patient comorbidities as (1) clinical discharge records report the ICD codes related only to the specific reason(s) of admission; (2) comorbidities are generally listed in clinical records which vary in form from hospital to hospital.

## Conclusions

The number of proximal femoral fractures is increasing both in Lombardy and in Italy, even if the age-adjusted incidence is decreasing. This is possibly due to effective prevention policies. In any case, the overall trend suggests that further efforts are needed to contrast the effect of population aging.

Two policies significantly impact the outcomes and the use of resources after proximal femoral fractures. First, the centralization of patients in high-volume hospitals demonstrated potential in reducing the risk of death during the 24 months after proximal femur primary fractures, and when patients were treated in these centres, the costs associated with primary hospitalization were significantly reduced. Second, the data presented in this study shows the large effect of an early intervention (< 48 h from hospitalization) in reducing the hazard of death in the 24 months after the event; respecting this timeframe reduced the costs associated with the primary fracture.

## Data Availability

The data that support the findings of this study are available from the Region of Lombardy but restrictions apply to the availability of these data, which were used under license for the current study, and so are not publicly available. Data are available from the Region of Lombardy upon request and permission, over which the authors of the present research have no decisional power.

## List of Abbreviations

CI: Confidence Interval
ICD-9-CM: International Classification of Disease, Ninth Revision, Clinical Modification
SE: Standard Error
SEM: Standard Error of the Mean
THA: Total Hip Arthroplasty
